# Increased cerebral *(R)*-[^11^C]PK11195 uptake and glutamate release in a rat model of traumatic brain injury: a longitudinal pilot study

**DOI:** 10.1186/1742-2094-8-67

**Published:** 2011-06-14

**Authors:** Hedy Folkersma, Jessica C Foster Dingley, Bart NM van Berckel, Annemieke Rozemuller, Ronald Boellaard, Marc C Huisman, Adriaan A Lammertsma, W Peter Vandertop, Carla FM Molthoff

**Affiliations:** 1 Neurosurgical Center Amsterdam, VU University Medical Center, De Boelelaan 1117, NL-1081 HV Amsterdam, the Netherlands; 2 Department of Nuclear Medicine & PET Research, VU University Medical Center, De Boelelaan 1117, NL-1081 HV Amsterdam, the Netherlands; 3 Department of Pathology, VU University Medical Center, De Boelelaan 1117, NL-1081 HV Amsterdam, the Netherlands

## Abstract

**Background:**

The aim of the present study was to investigate microglia activation over time following traumatic brain injury (TBI) and to relate these findings to glutamate release.

**Procedures:**

Sequential dynamic *(R)*-[^11^C]PK11195 PET scans were performed in rats 24 hours before (baseline), and one and ten days after TBI using controlled cortical impact, or a sham procedure. Extracellular fluid (ECF) glutamate concentrations were measured using cerebral microdialysis. Brains were processed for histopathology and (immuno)-histochemistry.

**Results:**

Ten days after TBI, *(R)*-[^11^C]PK11195 binding was significantly increased in TBI rats compared with both baseline values and sham controls (p < 0.05). ECF glutamate values were increased immediately after TBI (27.6 ± 14.0 μmol·L^-1^) as compared with the sham procedure (6.4 ± 3.6 μmol·L^-1^). Significant differences were found between TBI and sham for ED-1, OX-6, GFAP, Perl's, and Fluoro-Jade B.

**Conclusions:**

Increased cerebral uptake of *(R)*-[^11^C]PK11195 ten days after TBI points to prolonged and ongoing activation of microglia. This activation followed a significant acute posttraumatic increase in ECF glutamate levels.

## Background

Microglia are immunocompetent brain cells, expressing a variety of cytokine, chemokine, and neurotransmitter receptors, including receptors for glutamate, the principle excitatory amino acid (EAA) in the central nervous system (CNS), when activated [1]. In the neuroinflammatory cascade that follows traumatic brain injury (TBI), activated microglia may play a crucial role in the aetiology and progression of excito-neurotoxic brain lesions. Excito-neurotoxicity after TBI mainly results from excessive glutamate release with subsequent excessive influx of Ca^2+^, primarily mediated by N-methyl D-aspartate (NMDA) glutamate receptors [2]. Glutamate release induces excitotoxicity and contributes to the pathophysiology of numerous neurological diseases including ischemia, inflammation, epilepsy, and neurodegenerative diseases [3,4]. In TBI, glutamate is released in large quantities into the extracellular fluid (ECF) by neurons and glial cells. However, in the posttraumatic neuroinflammatory cascade the relation between initial glutamate release and time course of microglia activation is not yet clear.

*In vivo*, activated microglia can be quantified using *(R)*-[^11^C]PK11195 (1-[2-chlorophenyl]-*N*-methyl-*N*-[1-methyl-propyl]-3-isoquinoline carboxamide) and positron emission tomography (PET) [5,6]. In CNS diseases, transition of microglia from a normal resting state into a pathologically activated state has been associated with a marked increase in expression of the 18 kDa translocator protein (TSPO), previously known as the peripheral-type benzodiazepine receptor [7,8]. *(R)*-[^11^C]PK11195 is a highly selective ligand for TSPO, which can be used to monitor distribution and time course of microglia activation following TBI [5].

The purpose of the present study was to investigate the feasibility of determining microglia activation over time following traumatic brain injury (TBI), to determine such activation, and to relate these findings to glutamate release. To this end, *in vivo *cerebral microdialysis after TBI or sham was combined with sequential *(R)*-[^11^C]PK11195 brain PET scans and autopsy.

## Methods

### Controlled cortical impact injury (CCI)

Wistar rats (male, body weight around 300 g, Harlan, The Netherlands) were either subjected to focal TBI (n = 6) or a sham procedure (n = 6). Anaesthetic procedures and surgical preparation for CCI have been described previously [9]. In short, rats were anaesthetized in a ventilated anaesthesia chamber. After endotracheal intubation with a 16-gauge Teflon catheter, rats were mechanically ventilated at a respiratory rate of 80 breaths per minute and remained under full anaesthesia during the whole experiment (Isofluran 1-2%, oxygen 0.45 volume %). The iliac artery was cannulated with a 14G polymer catheter (length 25 mm) and connected to a pressure transducer to monitor blood pressure and obtain blood gases. Vital signs were monitored continuously and level of anaesthesia was evaluated by monitoring the pinch reflex. Body temperature of the animals was kept within normal range (37 -38 °C) using cooling and heating pads. Animals were placed in a stereotactic frame, and a craniotomy was performed over the right parietal cortex between lambda and bregma and approximately 2 mm lateral to the central suture. A rounded impactor tip with a diameter of 7 mm was adjusted to produce an impact at the centre of the exposed brain with an impact velocity of 5 m·s^-1^, duration of 130 ms, and an impact depth of 3 mm. Sham-operated rats underwent identical surgical procedures, except for the CCI.

### (R)-[^11^C]PK11195 PET

*(R)*-[^11^C]PK11195 was synthesized according to the method described by Shah et al [10]. This procedure resulted in a GMP compliant, pyrogen-free, sterile batch of *(R)*-[^11^C]PK11195 with radiochemical purity of > 98% and mean specific activity of 100 ± 36 GBq·μmol^-1^. Rats were anaesthetized with Isofluran 5% in a ventilated anaesthesia chamber, before breathing through a ventilation mask (Isofluran 1-2%, oxygen 0.45 volume %) during scanning. *(R)*-[^11^C]PK11195 was administered via a jugular vein cannula. In all rats *(R)*-[^11^C]PK11195 brain scans were performed 24 hours before intervention (baseline), and at one and ten days after TBI.

PET studies were performed using a single lutetium oxy-orthosilicate (LSO) crystal layer High Resolution Research Tomograph (HRRT, CTI/Siemens, Knoxville, TN, USA). The spatial resolution of this scanner is approximately 2.5 mm in all directions, and the absolute point-source sensitivity is as high as 7%. Performance characteristics of this scanner have been reported previously [11]. The combination of high spatial resolution and high sensitivity makes the HRRT an ideal PET scanner for dynamic studies in small laboratory animals [12,13].

First, a transmission scan was acquired using a 740 MBq ^137^Cs point source to correct for photon attenuation. Subsequently, following injection of *(R)*-[^11^C]PK11195, a dynamic emission scan with a total duration of 90 minutes was acquired. All data were acquired in 64-bits list mode. After acquisition, list mode data were converted into 16 sinograms with frame durations increasing from 15 up to 300 s and reconstructed using a three-dimensional ordered set expectation maximization (OSEM 3-D) algorithm with a matrix size of 256 × 256 × 207, resulting in a cubic voxel size of 1.21 × 1.21 × 1.21 mm^3^. Administered *(R)*-[^11^C]PK11195 activity (15.8 ± 3.4 MBq/rat) and mass (0.15 ± 0.03 nmol/rat) was not significantly different between TBI and control rats.

### Microdialysis

**Figure 1:**
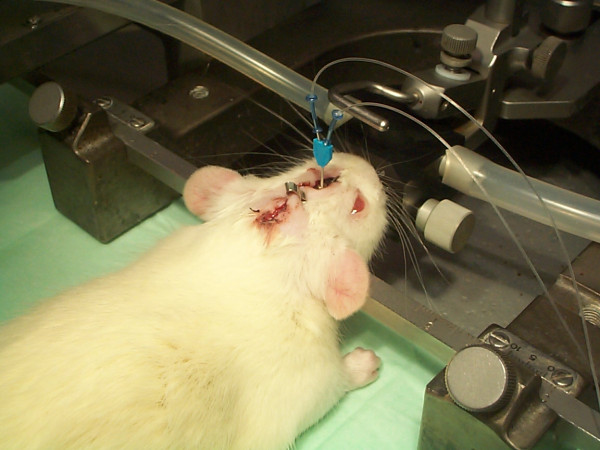
**Microdialysis catheter inserted into the brain parenchyma of an anaesthetized rat**.

PET and glutamate measurements were conducted in the same groups of animals. Immediately after CCI or sham procedure, in all rats a 2 mm-length microdialysis probe (CMA/12 14/02 PES CMA/Microdialysis, AB Solna, Sweden) with an outer diameter of 0.5 mm and a molecular weight cut-off of 100 kDa, was placed stereotactically at a depth of 3 mm into the surrounding zone of the lesion or normal brain parenchyma (controls) according to the following coordinates: -1.5 mm from bregma, 3.0 mm lateral from midsagittal line. The inlet (12 cm) and outlet (7 cm) tubings from the microdialysis probe were connected to a syringe cannula and a microfraction collector, respectively. Exactly 5 minutes after CCI or sham, perfusion of the semi-permeable system with artificial cerebrospinal fluid was started at a flow rate of 1.0 μl·min^-1 ^(Figure 1). Five sequential dialysate samples, at intervals of 60 minutes were collected and analyzed for glutamate using a CMA 600 analyzer (CMA/Microdialysis, Solna, Sweden).

Animals remained in the stereotactic frame throughout the experiment. Vital signs were monitored continuously and the level of anaesthesia was evaluated by monitoring the pinch reflex every 15 minutes. Body temperatures of the animals were kept within normal range (37 -38 °C) using cooling and heating pads. At the end of the experiment, the microdialysis probe was removed, the scalp incision closed and the rat removed from the stereotactic frame. After discontinuation of anaesthesia, rats were extubated when breathing spontaneously and monitored until full recovery.

Approval of the institutional animal ethics committee was obtained and all experiments were carried out in accordance with both the Dutch Law on Animal Experimentation and guidelines of the institutional committee on animal experimentation.

### Brain tissue preparation

Ten days after the experiment, the entire brain was removed promptly from the skull after decapitation. All brains were fixed in 4% buffered formalin, processed and embedded in paraffin according to routine procedures. Fixed axial tissue sections of 8 μm were mounted on coated slides and dried overnight at 37°C. Based on routine HE staining of all sections throughout the brain, selected slices of the lesion or sham region with an extra margin of 2 mm around this area were selected for (immuno)histochemical analysis.

### (Immuno)histochemistry

Brain sections were immunohistochemically processed using appropriate primary antibodies: ED1 (1:100; mouse anti-rat lysosomal enzyme; Hycult Biotechnology, Uden, The Netherlands), GFAP (anti Ga5 1:200, Bioconnect, Huissen. The Netherlands), or OX-6 (1:200; mouse anti-rat MHC class II; AbD Serotec, Oxford, UK). After ED1 and GFAP staining, the Envision kit detection method (DAKO, Glostrup, DK) was used. For antibody OX-6, the Streptavidin-Biotin-horseradish peroxidase complex detection method was used (1:500, DAKO, Glostrup, DK). Negative controls were included by replacement of the primary antibodies with 1% bovine serum albumin/phosphate-buffered saline. Finally, visualization was performed using 0.02% 3.3'-diaminobenzidine tetrahydrochloride. Subsequently, sections were counterstained with haematoxylin, dehydrated and mounted.

For histomorphological analysis of injury and inflammatory cells, routine haematoxylin and eosin (H&E) staining was performed. Additionally, a routine Perl's iron staining was applied to identify the location of posttraumatic haemorrhages. An anionic fluorochrome, Fluoro-Jade B, was used as a marker for neuronal injury, delineating both degenerating neuronal cell bodies and its processes [14,15]. Briefly, after deparaffination and dehydration, sections were rinsed in distilled H_2_O and incubated at room temperature for 25 min in 0.06% potassium permanganate to ensure background suppression. After washing in distilled H_2_O, sections were incubated for another 30 min in a freshly prepared 0.001% Fluoro-Jade B solution. Finally, sections were washed thoroughly in distilled H_2_O, dried for one hour at 37 °C, dipped in xylene and mounted.

A pathologist (AJR) and two investigators (JCFD; CFMM) scored the ED-1, OX-6, Perl's and Fluoro-Jade B results. Scoring was divided into no, moderate and strong positivity. In addition, the size of positive areas was measured for ED-1 and OX-6. GFAP staining was assessed for increased density of GFAP-positive astrocytes at the ipsilateral compared to the contralateral side.

### Image analysis

Time activity curves (TACs) of *(R)*-[^11^C]PK11195, reflecting delivery of the tracer to the brain and binding of the tracer to TSPO (right and left hemispheres), were derived for predefined volume of interest (VOI), drawn and copied from a standardized rat brain MRI, corrected for injected dose and animal weight, using the software package AMIDE (Amide 0.9.1 http://amide.sourceforge.net). For every rat the injected dose was derived from a VOI encompassing the total image. The TAC for this VOI showed a maximum value in the initial frames, after which a decrease was observed due to accumulation into the bladder. The shape of various total image TACs was similar, allowing a good estimate of the injected dose from the maximum value of these curves.

### Statistical Analysis

Statistical testing of areas under the curve (AUC) between baseline TACs and TACs after TBI or sham were performed using a one tailed Student's t-test, testing the a-priori hypothesis that (*R)*-[^11^C]PK11195 will be increased after TBI. Microdialysis data are expressed as mean ± standard deviation. Statistical significance of differences in ECF glutamate between TBI rats and controls at all time points was assessed using a one-tailed Student's t-test. Relationships between sham and TBI for all stainings were calculated using Spearman's correlation statistics. Because of non-normal distribution and differences in homogeneity of variances in protein expression levels (OX-6, ED-1, Perl's and Fluoro-Jade B) and lesion size (OX-6, ED-1 and Perl's), as assessed by the Kolmogorov-Smirnov test of normality and the Levenes test, statistical evaluation was performed using the non-parametric Mann-Whitney U test. P-values < 0.05 were considered statistically significant (SPSS version 15.0.1, SPSS Inc., Chicago, IL, USA).

## Results

### (R)-[^11^C]PK11195 PET

**Figure 2:**
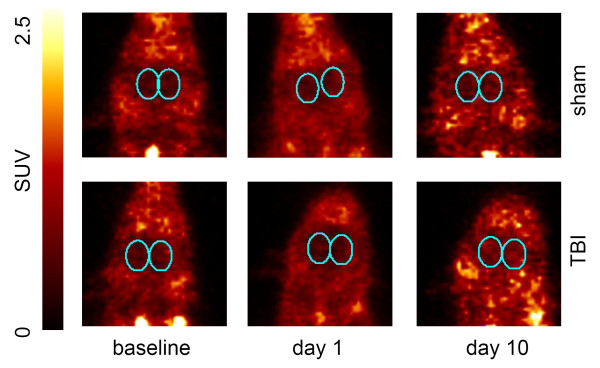
**Representative examples of summed PET images showing uptake of *(R)*-[^11^C]PK11195 in rat brain at baseline and at one and ten days after TBI or sham**.

**Figure 3:**
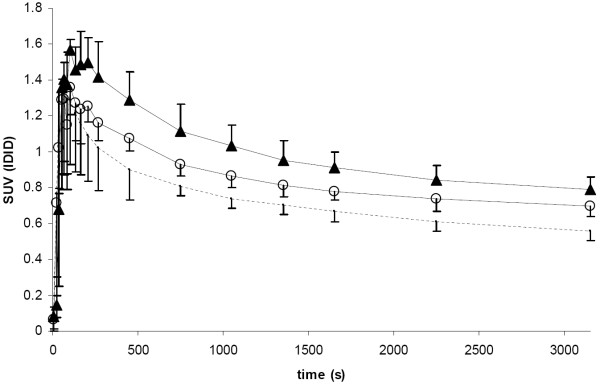
**Mean SUV of the image-derived injection dose (IDID) with error bars over time for baseline (--), sham (О) and TBI (▴) animals at 10 days**.

In total, 28 PET scans were performed and 21 *(R)*-[^11^C]PK11195 scans (9 baseline, 8 at day one (4 CCI, 4 sham), 4 at day 10 (2 CCI, 2 sham) were available for further analysis. Two rats died after the baseline scan, two rats died shortly after CCI and sham procedure due to cardiac arrest, and another four rats died before day ten. Representative examples of summed PET images of baseline, one and ten days after TBI or sham procedure are shown in Figure 2. One day after surgery, no statistically significant differences in *(R)*-[^11^C]PK11195 brain uptake were found between TBI and control rats, nor compared to baseline values. Ten days after TBI, however, an increase in cerebral uptake (SUV) of *(R)*-[^11^C]PK11195 was statistically significant in TBI rats (0.79 ± 0.07) compared with both corresponding baseline values (0.56 ± 0.05, p = 0.006) and sham-operated animals (0.70 ± 0.06, p = 0.026) (Figure 3).

### In vivo microdialysis

**Figure 4:**
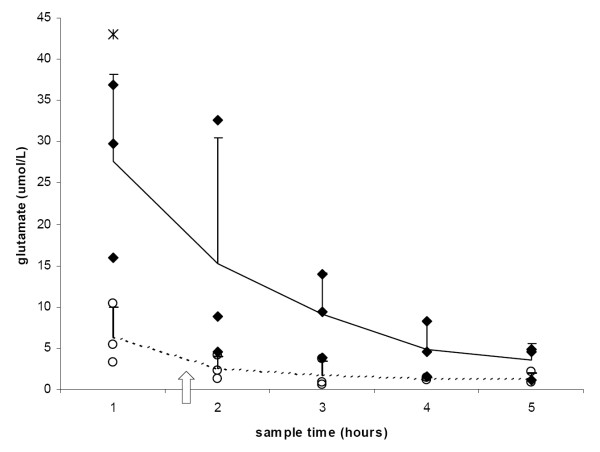
Dialysate glutamate concentrations as a function of time following CCI (♦ n = 3) and sham (о n = 3) (* p < 0.05). Vertical lines represent SD. The arrow indicates baseline ECF glutamate release (sham).

Accurate microdialysis samples were obtained for six rats, three TBI and three sham (controls). In two rats, sampling failed because of leakage of the microdialysis probe. ECF glutamate values one hour after TBI were significantly higher than corresponding values in sham rats (27.6 ± 10.6 μmol·L^-1 ^and 6.4 ± 3.6 μmol·L^-1^, respectively; p < 0.05). Moreover, at all time points mean glutamate levels in TBI rats were higher than in sham controls (Figure 4).

### Immunohistochemistry

**Figure 5:**
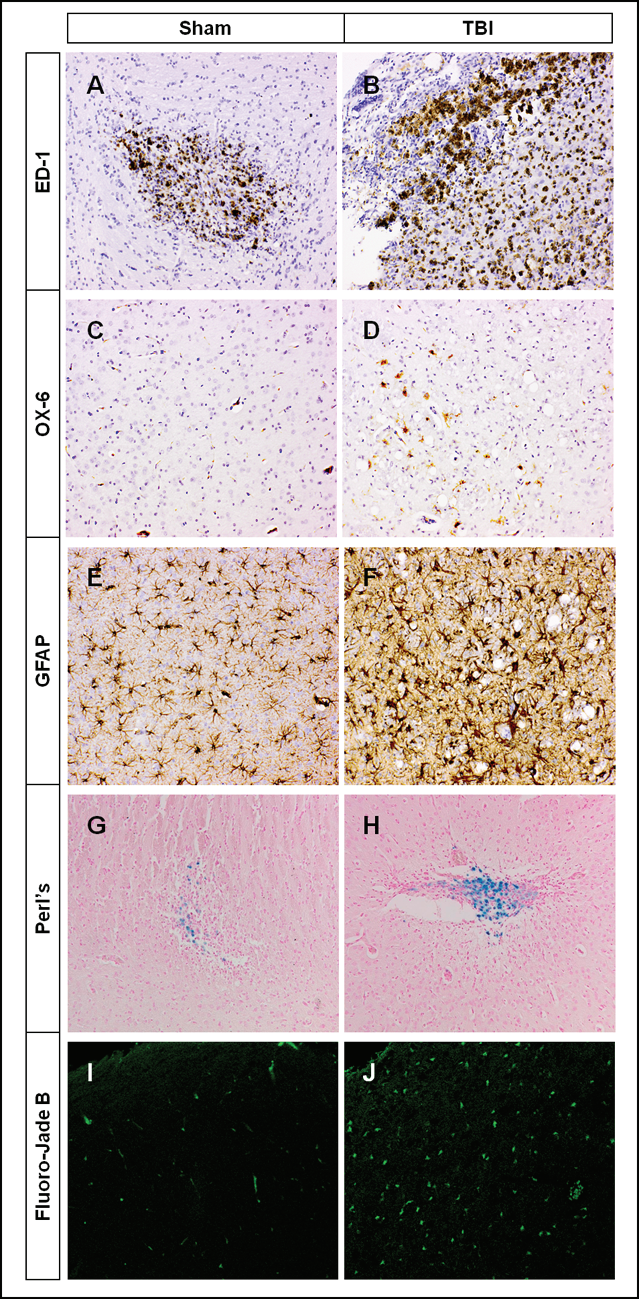
**Representative images of (immuno)histochemical staining of brain tissue (TBI and control) with ED-1, OX-6, GFAP, Perl's, and Fluoro-Jade B ten days after the microdialysis experiment**.

Representative examples of brain tissue stained for ED-1, OX-6, GFAP, Perl's, and Fluoro-Jade B are shown in Figure 5. The morphology of ED-1-positive cells clearly indicates highly activated microglia and/or macrophages. ED-1-positive cells were present in both TBI and sham-operated rats, but the size of the area of positive ED-1 staining was larger in the ipsilateral hemisphere of TBI rats, compared to both contralateral hemisphere and sham-operated rats (Figure 5 A+B). OX-6 staining was present predominantly in the ipsilateral hemisphere of TBI rats. It was minimal in the contralateral hemisphere and both hemispheres of sham-operated rats. OX-6-positive cells were morphologically identified as activated microglia. The size of stained areas markedly differed between injured and non-injured brain tissue (Figure 5 C+D). GFAP expression in the injured hemisphere was increased compared with that in the contralateral hemisphere. In sham-operated rats, the density of astrocytes was homogeneous over the entire brain (Figure 5 E+F). Perl's staining, indicative of haemorrhages, was clearly increased in size and intensity in the injured hemisphere of TBI rats (Figure 5 G+H). In TBI rats, Fluoro Jade B positivity was exclusively seen in the ipsilateral hemisphere of TBI rats (r = 0.83, p < 0.02). No Fluoro Jade B staining, indicating degenerating neurons was found in the contralateral hemisphere nor in sham brain tissue (Figure 5 I+J).

## Discussion

This study demonstrates the technical feasibility of TSPO imaging in experimental TBI. Correlations over time were investigated between posttraumatic microglia activation, determined by sequential *(R)*-[^11^C]PK11195 scans, ECF glutamate release, measured with *in vivo *cerebral microdialysis, and (immuno)-histopathology in a rat model of TBI. An increase was found in *(R)*-[^11^C]PK11195 cerebral uptake ten days after TBI. Glutamate concentrations were increased one hour after TBI compared to those after sham procedure.

To acquire pharmacologically and physiologically accurate ligand-receptor binding data in small animals using HRRT, a high specific activity is required to maintain a low degree of receptor occupancy [16]. The injected mass (0.15 ± 0.03 nmol/rat) in our study did not exceed the limits of site occupancy and is considered to be a tracer dosage. The significant increase in *(R)*-[^11^C]PK11195 uptake after TBI is in line with results of Toyama et al. [17], who found increased *(R)*-[^11^C]PK11195 binding in the injured site three days after an ethanol brain injury. Ito et al. [18] found significant differences in distribution volumes as well as an increased expression of inflammatory cytokines between injured and controls rats in specific brain areas four days after injury. Recently, Yu et al. [19] found a more-than-3-fold increase in [^18^F]fluoroethyl-DAA1106 uptake in traumatized brain tissue compared to controls, peaking one week after lateral fluid percussion brain injury. A relatively rapid accumulation of phagocytic microglia and macrophages at the necrotic core was reported, followed by increased TSPO expression. Although ECF glutamate was not measured, those findings are in line with the present results.

Peak ECF glutamate concentrations were found in the first hour of the microdialysis experiment. In TBI rats, this peak concentration was significantly higher than in controls. Over subsequent hours, glutamate concentrations gradually declined in both groups, but remained higher in TBI rats than in sham rats. When data from cerebral microdialysis experiments are analysed, the component resulting from the surgical procedure itself has to be taken into account. By penetrating brain parenchyma, the microdialysis catheter could give rise to a focal brain injury [20]. Haemostatic and local environmental disturbances caused by this penetration might result in temporary alterations in glutamate concentrations in ECF. Woodroofe et al. [20] showed that, without additional trauma, microdialysis catheters gave rise to a local immune response in brain parenchyma two days after implantation. Pathological excessive glutamate release, exceeding excitotoxic thresholds, results in activation of postsynaptic ionotropic and metabotropic receptors [21]. Activation of non-N-methyl-D-aspartate (non-NMDA) glutamate receptors generates an influx of monovalent cations (Na^+ ^and K^+^) followed by a passive influx of water, inducing cytotoxic oedema [22]. Activation of NMDA glutamate receptors elicit an excessive influx of calcium, stimulating activation of phospholipases and generation of oxygen-derived free radicals [2]. Taken together, this detrimental state of excitotoxicity results in apoptosis of resident cells of the brain [23,24]. Consequently, NMDA antagonists may be beneficial in preventing progressive brain injury after trauma [25]. Indeed, efficacy of NMDA antagonists has been demonstrated in several preclinical brain injury studies. For instance, NMDA antagonists may be beneficial in preventing secondary neuronal damage by ischemia, neurological motor dysfunction, impairment in spatial memory and focal brain edema at the site of injury. Furthermore, they may affect synaptic plasticity [26-28]. In addition, in situ administration of kynurenic acid, an EAA antagonist and specific NMDA receptor blocker, attenuates rapid microglial and astroglial reactions in rat hippocampus following TBI [29]. Results of the present study underline that increased ECF glutamate concentrations in the acute posttraumatic phase precede increased microglial activation in traumatized brain tissue [30]. Future studies should address whether there is a beneficial effect of EAA antagonists on late-phase microglia activation.

Microglial activation and brain injury were assessed by immunohistochemical and Fluoro Jade B staining. From a morphological point of view, ED-1-positive cells are activated macrophages and/or highly activated microglia. The exclusive site where ED-1-positive cells were present was at the ipsilateral hemisphere of TBI rats. This is in accordance with a study by Lemstra et al. [31], who showed that CD68 is a lysosomal membrane marker and predominantly stains microglia in a highly active, phagocytic state. OX-6 is another suitable marker of activated microglia, and OX-6 staining demonstrated a clear difference between sham and TBI rats. The present results indicate that both density and area of OX-6-positive cells are higher at the site of injury. These findings indicate that activated microglia have migrated to the site of injury ten days after TBI which is in accordance with studies by Cho et al [32]. In TBI, Fluoro-Jade B is a very effective marker for locating degenerating neurons [14]. In the present study, Fluoro-Jade B-positive cells were only seen in the injured hemisphere. These findings, together with neuronal tissue damage and microglia activation at the site of the injury confirm increased *(R)*-[^11^C]PK11195 binding in the injured hemisphere in TBI rats, ten days after CCI.

## Conclusions

Significantly increased cerebral uptake of *(R)*-[^11^C]PK11195 ten days after TBI points to prolonged and ongoing activation of microglia. This increased posttraumatic microglia activation follows an acute posttraumatic ECF glutamate release.

## Competing interests

The authors declare that they have no competing interests.

## Authors' contributions

HF, BNMB, AAL, WPV and CFMM participated in the design of the study, HF drafted the manuscript, and HF and CFMM carried out the experimental microdialysis and PET studies (technical support MG). HF and JCFD performed the statistical analysis (statistical support PK), JCFD carried out the immunoassays, and AR interpreted the immunoassays. MCH helped in analyzing the PET data. BNMB, RB, AAL, WPV, and CFMM critically revised the manuscript. All authors read and approved the final manuscript. Contributors who do not meet the criteria for authorship (MG, PK) are listed in the acknowledgements section.
